# Shift Work and Acute Coronary Syndrome: Mechanisms, Modifying Factors, and Clinical Prevention

**DOI:** 10.3390/healthcare14213651

**Published:** 2026-10-28

**Authors:** Luling Wang, Kumar Ganesan, Xing Liu, Lili Yu, Qiu Chen

**Affiliations:** 1 Medical Sciences Division, Faculty of Chinese Medicine, Macau University of Science and Technology, E101, Block E, Avenida Wai Long, Taipa, Macao 999078, China; 2 School of Chinese Medicine, LKS Faculty of Medicine, The University of Hong Kong, Hong Kong, China; 3 Science and Technology Innovation Center, Shanghai Municipal Hospital of Traditional Chinese Medicine, Shanghai University of Traditional Chinese Medicine, Shanghai 200071, China; 4 Department of Endocrinology, Hospital of Chengdu University of Traditional Chinese Medicine, Chengdu 610072, China

**Keywords:** shift work, acute coronary syndrome, circadian misalignment, sleep, occupational health

## Abstract

**Background/Objectives:** Shift work causes recurrent circadian misalignment. This review synthesizes evidence linking shift work to acute coronary syndrome (ACS) and proposes a framework for prevention, return-to-work decisions, and future research. **Methods:** We conducted a narrative review using targeted searches of PubMed and major guideline repositories through July 2026. Search terms included shift work, night work, acute coronary syndrome, myocardial infarction, coronary heart disease, circadian misalignment, sleep, thrombosis, and cardiovascular risk. Revision-stage verification also examined atherosclerosis, coronary plaque, plaque vulnerability, and ischemia. Priority was given to prospective cohorts, systematic reviews, meta-analyses, controlled human studies, consensus statements, and major guidelines. **Results:** Night and rotating shift work are associated with a modestly increased risk of coronary heart disease (relative risk ~1.2–1.3), with duration-response gradients. Direct ACS-specific evidence is more limited but consistent with increased incidence and recurrence. Mechanistically, circadian misalignment and sleep deficiency promote autonomic dysregulation, hypertension, insulin resistance, dyslipidemia, inflammation, endothelial dysfunction, and atherosclerosis. Acute autonomic, hemodynamic, and prothrombotic changes may trigger plaque disruption or supply-demand ischemia. Risk modifiers include schedule intensity, cumulative exposure, chronotype, age, sex, comorbidities, sleep disorders, occupation, and health behaviors. Pregnancy is a specific evidence gap. **Conclusions:** Night and rotating shift work are probable contributory risk factors for ACS in susceptible workers. Clinical practice should integrate work schedule and sleep health into cardiovascular assessment, especially after ACS. Roster redesign offers the most defensible preventive strategy. Evidence that circadian interventions prevent ACS remains insufficient. More prospective ACS-specific studies are needed.

## 1. Introduction

Acute coronary syndrome (ACS) includes ST-segment-elevation myocardial infarction, non-ST-segment-elevation myocardial infarction, and unstable angina. Atherothrombotic type 1 myocardial infarction should be distinguished from type 2 myocardial infarction caused by an imbalance between myocardial oxygen supply and demand [1,2]. Conventional risk factors account for much of the global coronary burden, but biological, behavioral, and social exposures interact in determining an individual’s risk [3,4]. Shift work is relevant because it can simultaneously alter sleep, circadian timing, diet, physical activity, stress, and access to preventive care.

Franz Halberg, widely regarded as the father of modern chronobiology and a pioneer of chronocardiology, helped establish quantitative biological-rhythm research through work in the 1950s. He introduced the term “circadian” in 1959 [5]. This historical perspective underpins the present distinction between endogenous biological timing and externally imposed work schedules.

Permanent nights, rotating shifts, early starts, irregular on-call duty, and extended shifts represent different exposures. Their biological effects depend on night-work frequency, rotation pattern, cumulative duration, intershift recovery, and the worker’s actual sleep and light exposure [6,7]. Shift-work disorder, characterized by schedule-related insomnia and/or excessive sleepiness, affects a subset of exposed workers. It illustrates the substantial sleep burden associated with nonstandard hours [8]. Thus, a binary shift-work classification cannot capture the biological dose of work timing.

The burden of shift work and cardiovascular disease is substantial. A large proportion of the global workforce is engaged in night and rotating schedules. Epidemiological studies consistently associate these schedules with increased cardiovascular risk. A systematic review of 34 studies involving more than two million participants reported a relative risk of 1.23 for myocardial infarction among shift workers [9]. A subsequent dose–response meta-analysis reported higher cardiovascular risk with longer exposure [10]. A 2025 meta-analysis of 23 cohorts and 3,340,377 participants reported pooled relative risks of 1.13 for cardiovascular disease incidence and 1.27 for cardiovascular disease mortality [11]. For incident coronary heart disease, the relative risk was 1.22 [11]. Each additional five years of night work was associated with a relative risk of 1.07 for cardiovascular disease incidence [11]. These findings establish shift work as a relevant occupational exposure for cardiovascular prevention.

**Figure 1:**
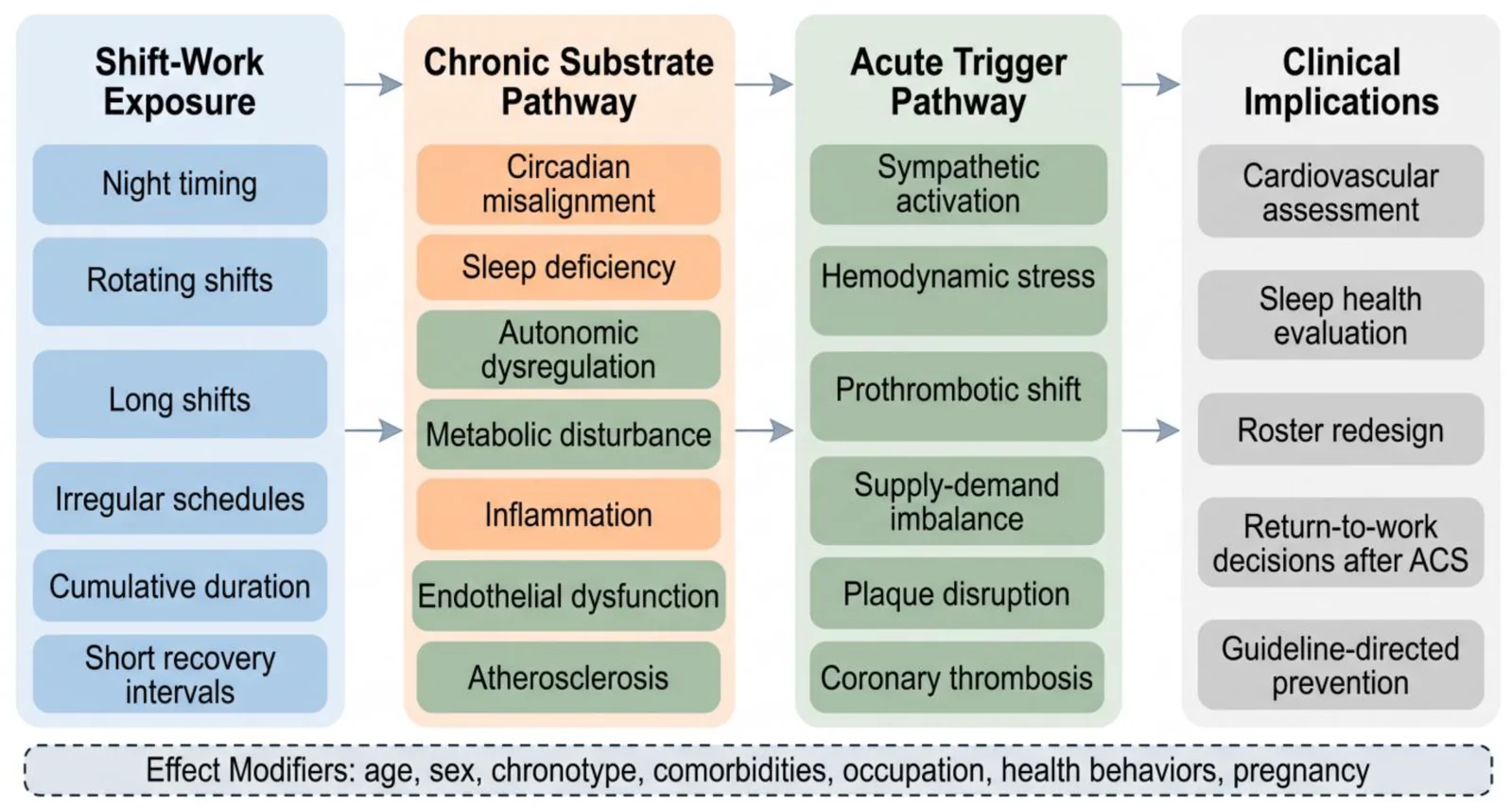
Conceptual framework linking shift work to acute coronary syndrome. This figure presents the epidemiology–mechanism–clinical implications framework used in this review. The left panel shows shift-work exposure dimensions, including night timing, rotation, shift length, cumulative duration, and recovery intervals. The center panel shows chronic substrate pathways, including circadian misalignment, sleep deficiency, autonomic dysregulation, metabolic disturbance, inflammation, endothelial dysfunction, and atherosclerosis. The right panel shows acute trigger pathways, including sympathetic activation, hemodynamic stress, prothrombotic shifts, and supply-demand imbalance. The bottom panel shows clinical implications, including cardiovascular assessment, sleep health evaluation, roster redesign, and return-to-work decisions after ACS. Effect modifiers, including age, sex, chronotype, comorbidities, occupation, and health behaviors, apply across all pathways. The figure is a conceptual synthesis, not a validated risk score. Abbreviations: ACS, acute coronary syndrome. [1,2,6,7,12,13,14,15,16,17,18,19,20,21,22,23,24,25,26,27,28,29].

The conceptual framework of this review distinguishes chronic substrate formation from acute triggering. Repeated circadian misalignment and sleep deficiency may promote autonomic dysregulation, hypertension, insulin resistance, dyslipidemia, inflammation, endothelial dysfunction, and atherosclerosis. These processes form a susceptible coronary substrate over years. Acute autonomic surges, hemodynamic changes, and prothrombotic shifts may then precipitate plaque disruption or supply-demand ischemia. This double-hit model is presented as an interpretive framework rather than a validated risk score. It organizes the evidence and identifies where uncertainty remains. Figure 1 summarizes this conceptual framework and the overall structure of the review.

Current ACS and cardiovascular prevention guidelines do not yet incorporate shift-work exposure into standard risk assessment [1,21]. This gap persists despite accumulating epidemiological and mechanistic evidence linking night work to coronary events. Direct ACS-specific evidence is more limited than evidence for broader cardiovascular outcomes and coronary heart disease. Prospective studies specifically designed to adjudicate ACS outcomes in shift workers remain scarce. The magnitude of an ACS-specific causal effect therefore remains uncertain.

This review asks what the evidence establishes about shift work and coronary events, which mechanisms plausibly connect exposure to these outcomes, and which clinical and workplace actions are proportionate to that evidence. The review first describes exposure definitions and epidemiological evidence. It then examines controlled human and intermediate-outcome evidence. It integrates these findings into a mechanistic framework. It discusses modifying factors, clinical implications, and return-to-work decisions after ACS. It concludes with limitations and prioritized research directions. This structure provides a clear direction for the subsequent sections.

## 2. Methods

### 2.1. Review Design, Information Sources, and Scope

This narrative review used an epidemiology–mechanism–clinical implications framework. PubMed was the primary database, supplemented by Embase, Cochrane Library, Web of Science, and major guideline databases (ESC, AHA/ACC, WHO), searched from database inception to 31 July 2026, with reference-list checking for foundational studies. PubMed was used for biomedical, occupational-health, cardiovascular, and sleep research; guideline sources supplied clinical recommendations that cannot be derived from individual observational studies alone. These complementary sources support a focused clinical synthesis but do not establish exhaustive coverage of the occupational or multidisciplinary literature.

The reported search terms included “shift work”, “night work”, “acute coronary syndrome”, “myocardial infarction”, “coronary heart disease”, “circadian misalignment”, “sleep”, “thrombosis”, and “cardiovascular risk”. Source checking also examined specified intervention papers and searched for atherosclerosis, coronary plaque, and ischemia in relation to shift work. This focused verification was not intended as a systematic search; it aimed to ensure that evidence supporting the proposed conceptual framework was sufficiently comprehensive. No separate supplementary search was performed for plaque vulnerability or disruption; this limitation is acknowledged in Section 4.4. Table 1 organizes shift-work exposure dimensions across reviewed studies. These dimensions are complementary and should not be reduced to a single yes/no variable [6,7].

ACS, acute coronary syndrome. The dimensions are complementary and should not be reduced to a single yes/no variable [6,7].

### 2.2. Inclusion and Exclusion Criteria

The revised synthesis covers adults undertaking night, rotating, irregular, or extended work schedules; patients with coronary disease are considered separately where relevant. Eligible evidence addresses incident or recurrent coronary events, broader cardiovascular outcomes, sleep/circadian disturbance, or cardiovascular and metabolic intermediates. Prospective cohorts, systematic reviews and meta-analyses, controlled human experiments, clinical guidelines, and consensus statements receive priority. Foundational observational studies and mechanistic reviews are retained where they explain the proposed pathways. Intervention evidence from non-shift-working populations is explicitly identified as indirect.

Reports unrelated to work timing, circadian biology, relevant cardiovascular mechanisms, or clinical management fall outside the scope. Duplicate reports do not count as independent evidence; overlapping cohorts and reviews are considered in interpreting consistency. Unverifiable quantitative claims are not retained as findings. Animal and tissue evidence informs biological interpretation but is not treated as clinical evidence of ACS prevention. Conflicting and null findings are retained. These criteria describe the scope of the revised narrative synthesis; they should not be represented as a prospectively registered selection protocol.

### 2.3. Independent Screening, Disagreement Resolution, and Reliability

Two reviewers (Luling Wang and Xing Liu) independently screened titles/abstracts and full texts. Disagreements were resolved by discussion; if disagreement persisted, Kumar Ganesan adjudicated. Cohen’s kappa was not calculated because independent pre-consensus decisions were not systematically archived; consensus decisions alone cannot recover independent agreement. This is acknowledged as a limitation.

### 2.4. Data Synthesis and Trustworthiness

Evidence was organized by study design, population, shift exposure, outcome, principal finding, and limitation. Results distinguish coronary/ACS outcomes from broader cardiovascular composites and intermediate biomarkers. Relative risks and hazard ratios retain their original outcome definitions and are not pooled because exposures, populations, and designs differ. Mechanistic observations are interpreted separately from causal claims about ACS [30].

Miles and Huberman’s qualitative quality criteria provide a framework for evaluating this synthesis [31]. Confirmability requires traceable links between claims and sources; dependability requires explicit selection and classification rules; credibility requires comparison across designs and retention of contradictory findings; transferability requires clear population and occupational boundaries; and utilization requires recommendations proportionate to evidence. The revised tables and separated Results/Discussion make these distinctions explicit. These criteria are not a numerical reliability test, and no unsupported percentage-agreement threshold is substituted for Cohen’s κappa. Mechanism-based inferences are identified using OCEBM 2011, with directness to ACS considered separately [32].

### 2.5. Use of Generative AI

**Figure 2:**
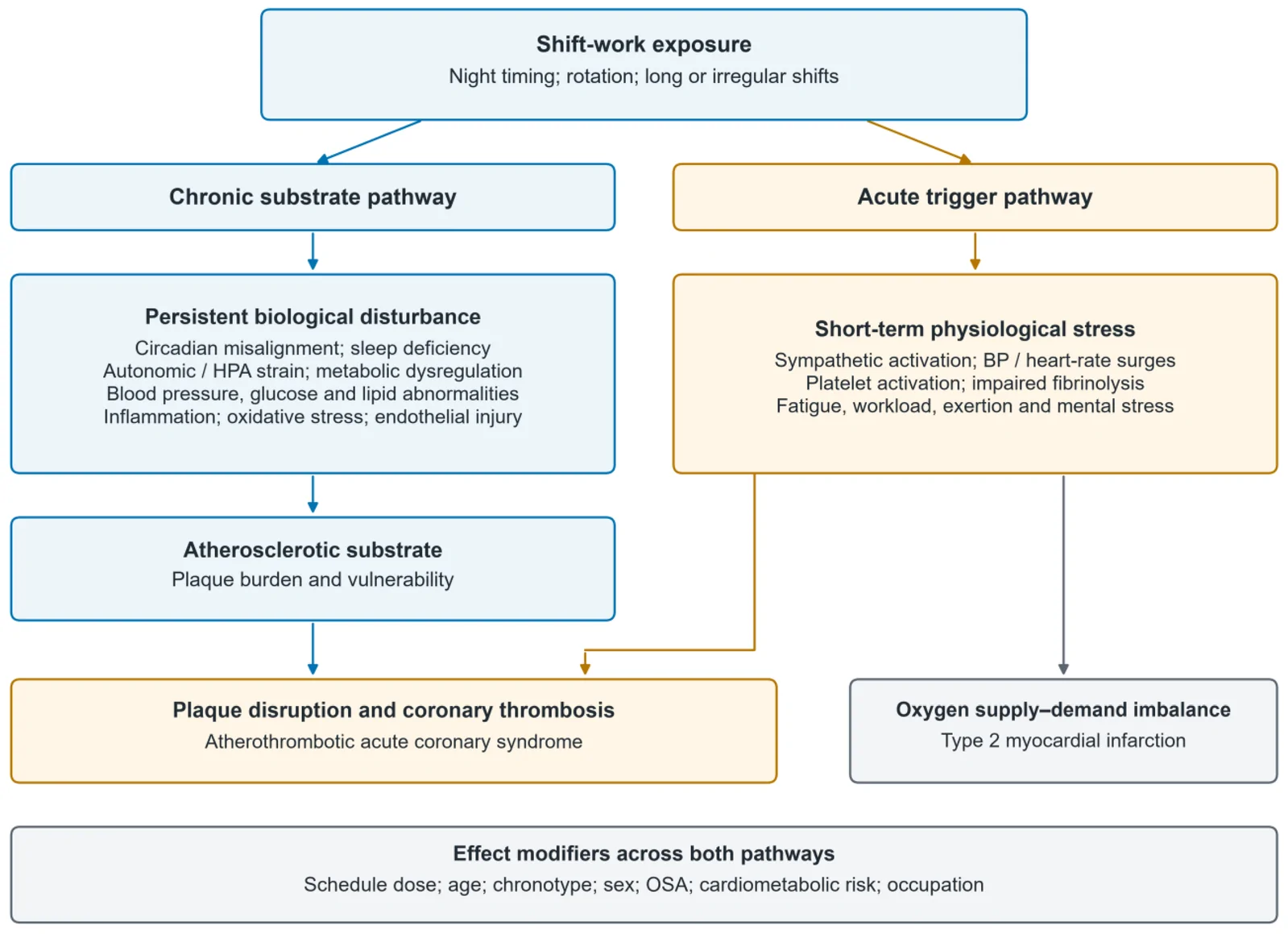
Proposed double-hit model of shift work and coronary events. Repeated metabolic, autonomic, inflammatory, and endothelial disturbances may increase coronary susceptibility, while acute hemodynamic and hemostatic stress may trigger events. Effect modifiers apply across pathways. The model is a conceptual synthesis, not a validated risk score. Abbreviations: ACS, acute coronary syndrome; BP, blood pressure; HPA, hypothalamic–pituitary–adrenal; MI, myocardial infarction; OSA, obstructive sleep apnea [1,2,12,13,14,15,16,17,18,19,20,21,22,23,24,25,26,27,28,29].

**Figure 3:**
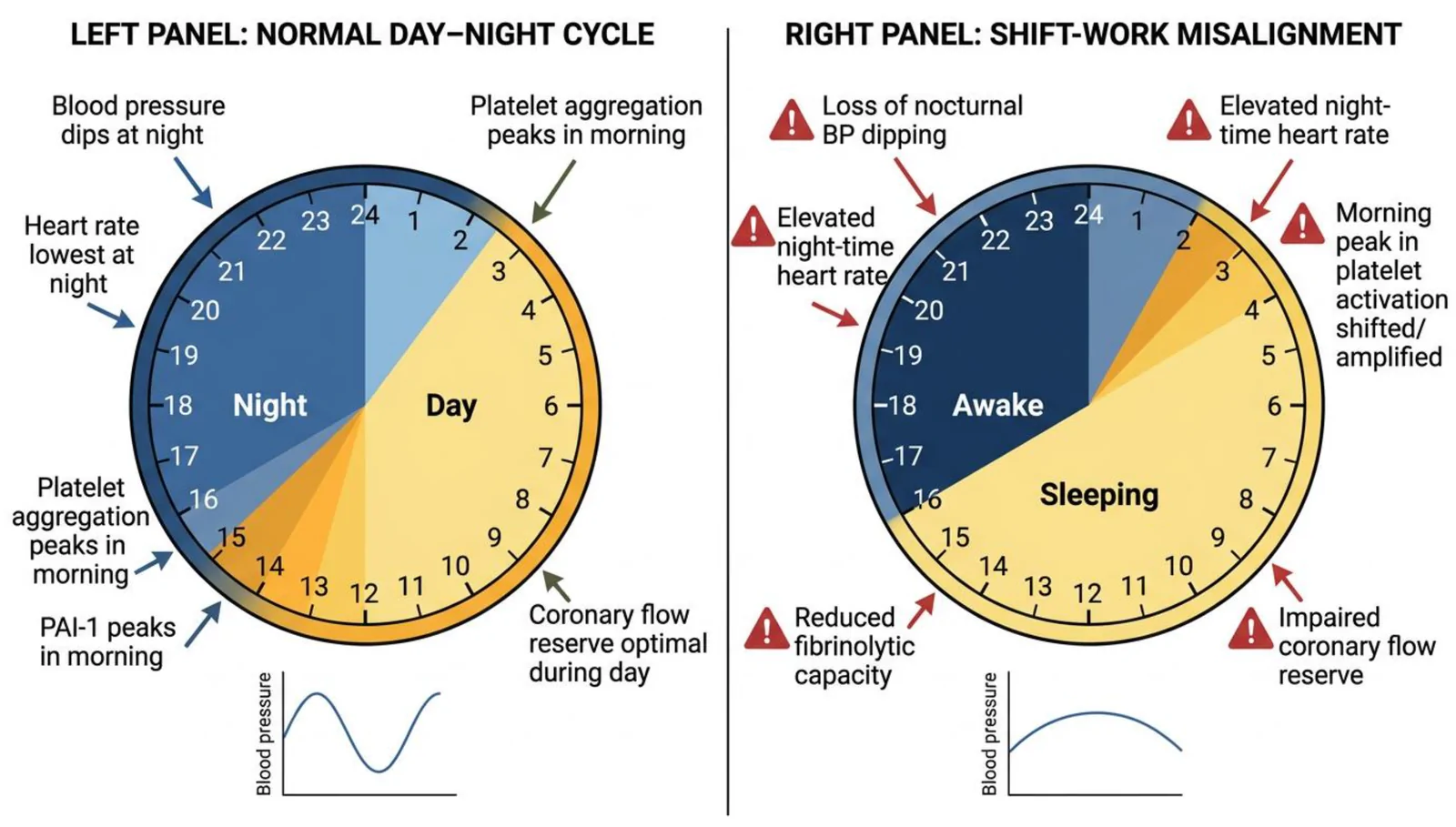
Circadian regulation of cardiovascular physiology and the impact of shift-work misalignment. (Left Panel) In individuals with a normal day–night cycle, physiological parameters exhibit predictable circadian rhythms: blood pressure and heart rate decline during nocturnal sleep; platelet aggregability and plasminogen activator inhibitor-1 (PAI-1) peak in the biological morning; and coronary flow reserve is optimized during daytime activity [16,17,18,19,23,24,25,26]. (Right Panel) In shift workers, exposure to nocturnal wakefulness, daytime sleep, and irregular meal timing disrupts these rhythms. Consequences include loss of nocturnal blood-pressure dipping, sustained sympathetic activation, blunted parasympathetic recovery, altered hemostatic profiles, and impaired myocardial oxygen supply–demand balance. These disruptions may precipitate acute coronary events in workers with pre-existing atherosclerotic substrate. Abbreviations: BP, blood pressure; PAI-1, plasminogen activator inhibitor-1.

**Figure 4:**
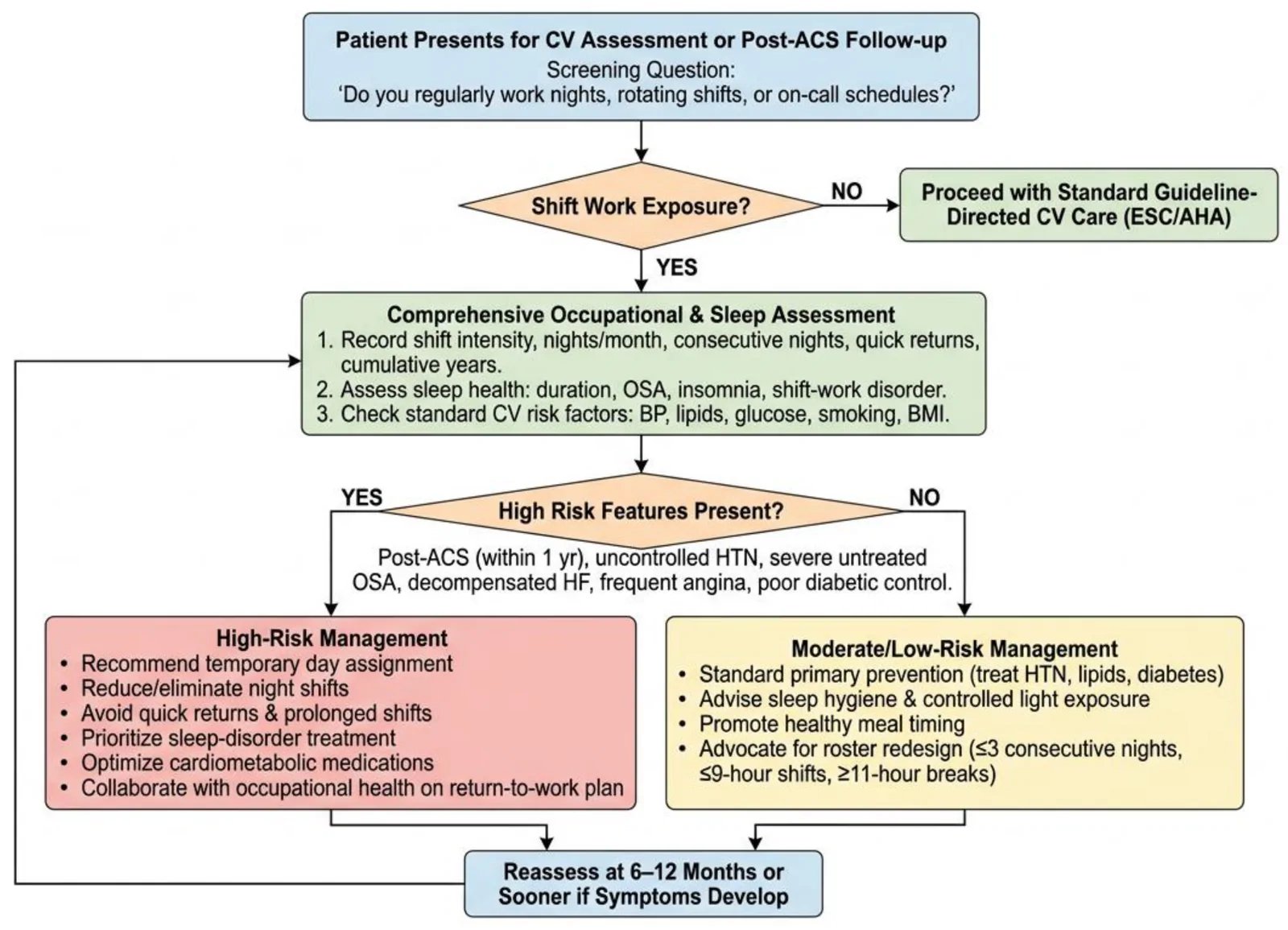
Proposed clinical algorithm for integrating shift-work exposure into cardiovascular risk assessment and management.

We used ChatGPT (OpenAI, GPT-4o, OpenAI, San Francisco, CA, USA) to assist in drafting summaries for tables and figures, and OpenAI Codex (OpenAI, San Francisco, CA, USA) to assist with language editing, restructuring, source checking, and Python (version 3.9.12, Python Software Foundation, Wilmington, DE, USA) code for redrawing Figure 1, Figure 2, Figure 3 and Figure 4. No participant data or screening-agreement statistics were generated by AI. AI-assisted source checking did not constitute independent human screening. The authors retain responsibility for verifying the manuscript, references, figures, and final scientific interpretation.

## 3. Results

### 3.1. Exposure Definitions and Outcomes Represented in the Evidence

The epidemiological literature used heterogeneous definitions of shift work. Some studies classified schedules by their overlap with overnight hours; others distinguished fixed nights, rotating shifts, or irregular work [6,7]. In the Nurses’ Health Studies, rotating night work generally required at least three nights monthly in addition to day or evening shifts [33,34]. Other cohorts classified two-shift or three-shift systems without comparable information on night frequency or recovery. Exposure assessment ranged from a single baseline questionnaire to repeated reports of cumulative duration. Table 1 organizes the exposure dimensions represented across these studies.

Clinical endpoints also differed. Studies reported myocardial infarction, coronary heart disease (CHD), ischemic heart disease mortality, broader cardiovascular disease (CVD) composites, and recurrent major coronary events [8,9,10,33,34,35,36,37,38,39,40,41,42,43,44,45,46,47]. The Korean study labeled its outcome ACS but combined myocardial infarction with angina pectoris, without restricting the latter to adjudicated unstable angina [36]. Controlled human studies assessed metabolic, inflammatory, autonomic, or hemostatic outcomes rather than coronary events [16,17,18,19,20,21,23,24,48,49,50,51]. These evidence groups are presented separately below, with representative epidemiological studies summarized in Table 2.

**Table 2:** Selected epidemiological evidence linking shift work with coronary and cardiovascular outcomes.

Study	Design and Outcome	Principal Finding	Interpretation
Xi et al., 2025 [11]	Meta-analysis of 23 cohorts; 3,340,377 participants	CVD incidence RR 1.13 (1.10–1.16); mortality 1.27 (1.18–1.36); CHD incidence 1.22 (1.16–1.28)	CVD incidence increased with duration; broad outcomes and heterogeneous schedules.
Vyas et al., 2012 [9]	Systematic review of 34 studies; vascular events	MI RR 1.23 (1.15–1.31)	Large observational evidence base; exposure and endpoint heterogeneity.
Torquati et al., 2018 [10]	Systematic review and dose–response meta-analysis	Higher CVD/CHD risk with longer exposure	Supports an exposure gradient; endpoints extend beyond ACS.
Kawachi et al., 1995 [33]	Prospective US nurse cohort; CHD	≥6 years of rotating nights: RR 1.51 (1.12–2.03)	Early prospective evidence in women.
Fujino et al., 2006 [41]	Prospective Japanese male cohort; IHD mortality	Higher mortality among rotating-shift workers	Association stronger among hypertensive men.
Vetter et al., 2016 [34]	Two prospective nurse cohorts; CHD	Longer rotating-night exposure associated with increased risk; attenuation after cessation	Suggests partial reversibility after exposure ends.
Jankowiak et al., 2016 [43]	Population study; subclinical atherosclerosis	Current/cumulative night work associated with adverse vascular measures	Associations partly explained by lifestyle and dispositional factors.
Hublin et al., 2010 [46]	22-year twin cohort; cardiovascular morbidity/mortality	No clear independent association after adjustment	Null findings retained.
Barger et al., 2017 [35]	13,026 post-ACS patients; median 2.5-year follow-up	Prior overnight-work history: major coronary events HR 1.15 (1.03–1.29)	Direct post-ACS evidence. Observational exposure within a trial.
Ryu et al., 2025 [36]	Prospective Korean cohort; 3696 participants; reported MI/angina	Shift work: adjusted HR 1.74 (1.20–2.53)	Combined MI/angina endpoint. Subtypes not distinguished.

Parentheses indicate 95% confidence intervals where supplied. Associations do not establish causation. Abbreviations: CHD, coronary heart disease; CVD, cardiovascular disease; HR, hazard ratio; IHD, ischemic heart disease; MI, myocardial infarction; RR, relative risk.

### 3.2. Coronary and Cardiovascular Associations

A systematic review of 34 studies involving more than two million participants reported a relative risk of 1.23 for myocardial infarction among shift workers [9]. Torquati et al. subsequently reported higher CVD risk and a duration-response association, with increasing risk after approximately five years of exposure [10]. The 2025 meta-analysis by Xi et al. included 23 cohorts and 3,340,377 participants [11]. Pooled relative risks were 1.13 (95% confidence interval(CI), 1.10–1.16) for CVD incidence and 1.27 (95% CI, 1.18–1.36) for CVD mortality. For incident CHD, the relative risk was 1.22 (95% CI, 1.16–1.28). Each additional five years of night work was associated with a relative risk of 1.07 (95% CI, 1.04–1.09) for CVD incidence. Associations were reported for fixed and rotating schedules and in both sexes. The authors rated certainty as low or very low across outcomes [11].

Prospective cohorts and case–control studies supplied coronary-specific evidence, although estimates varied. Early Scandinavian studies reported associations with ischemic heart disease and myocardial infarction [38,39]. A population study also associated shift-work exposure with atherosclerosis and previous myocardial infarction [40]. Among 79,109 U.S. nurses, at least six years of rotating night work was associated with a 51% higher CHD risk than no rotating night work [33]. The Japanese Collaborative Cohort Study reported elevated ischemic heart disease mortality among rotating workers, including a stronger association among hypertensive men [41]. The Helsinki Heart Study reported an adjusted coronary risk of approximately 1.4 for shift workers [42]. In two later Nurses’ Health Study cohorts, longer rotating-night exposure was associated with a small absolute increase in CHD; associations weakened with increasing time since cessation [34].

Several studies reported null or attenuated estimates. A Finnish twin cohort found no clear elevation in cardiovascular morbidity after adjustment over 22 years [46]. Associations in a multi-ethnic Singapore cohort weakened after adjustment for socioeconomic and work-related characteristics [44]. The Gutenberg Health Study reported associations between night work and subclinical vascular measures that were partly explained by lifestyle and dispositional factors [43]. Shift work was also associated with cardiometabolic multimorbidity among people with hypertension, an outcome broader than coronary disease [45]. Reviews of coronary risk documented substantial differences in study populations, adjustment, and exposure measurement [37,47].

### 3.3. Incident MI/Angina and Coronary Events After ACS

In the Korean Genome and Epidemiology Study, 3696 participants, including 221 shift workers, contributed to the analysis [33]. There were 33 MI/angina events among shift workers and 277 among non-shift workers. The fully adjusted hazard ratio was 1.74 (95% CI, 1.20–2.53). Exposure was recorded through a baseline question about shift work involving nights. A smoking-stratified analysis found an association among non-current smokers but not current smokers; no interaction with alcohol consumption was reported [36]. This study provided prospective evidence for the combined MI/angina endpoint but did not distinguish coronary event subtypes.

Barger et al. examined sleep and occupational history in 13,026 patients enrolled within 30 days of ACS in SOLID-TIMI 52 [35]. A history of at least one year working three or more overnight shifts weekly was associated with subsequent major coronary events. The adjusted hazard ratio was 1.15 (95% CI, 1.03–1.29) over a median of 2.5 years. This composite comprised CHD death, myocardial infarction, or urgent coronary revascularization. Short sleep and a positive screening result for obstructive sleep apnea (OSA) were also associated with adverse outcomes. Shift-work exposure was observational within a randomized drug trial and described previous work history; it did not establish whether patients continued night work after ACS [35].

### 3.4. Controlled Human and Intermediate-Outcome Evidence

Laboratory protocols separated selected effects of circadian timing from habitual occupational conditions. In a forced-desynchrony experiment, misalignment reduced leptin, increased glucose despite higher insulin, raised mean arterial pressure, and reversed the usual cortisol profile [19]. A simulated night-work protocol increased blood pressure and inflammatory markers and altered autonomic regulation [16]. Experimentally imposed misalignment in chronic shift workers similarly increased C-reactive protein and blood pressure [17]. Autonomic recordings in rotating workers documented changes in cardiac autonomic profiles across work schedules [18]. These studies measured short-term physiological responses rather than ACS incidence.

Metabolic experiments reported effects across several complementary protocols. Prolonged sleep restriction combined with circadian disruption reduced resting metabolic rate and impaired insulin secretion [20]. Misalignment augmented insulin resistance and inflammatory responses under experimentally controlled sleep conditions [21]. Simulated night work reduced energy expenditure [48], while another protocol distinguished effects of circadian phase and misalignment on glucose tolerance [49]. Glucose handling differed across rotational work conditions in healthcare workers [50], and four days of simulated shift work reduced insulin sensitivity [51]. Differences in sleep opportunity, meal timing, participant characteristics, and protocol duration limited direct comparison of effect magnitudes.

Observational studies identified corresponding cardiometabolic outcomes. A meta-analysis linked rotating or night work with hypertension [52], and longitudinal studies in Japanese men reported blood-pressure progression with shift exposure [53,54]. Other meta-analyses reported associations with dyslipidemia, metabolic syndrome, obesity, and diabetes [55,56,57,58]. In female nurse cohorts, rotating night work and unhealthy lifestyle were jointly associated with increased diabetes risk [59]. These studies did not determine what proportion of the coronary association was mediated by each metabolic condition.

Vascular and inflammatory evidence was less uniform. Studies of shift workers reported subclinical atherosclerosis associations [40,43], while a systematic review found generally adverse oxidative-stress profiles across heterogeneous assays and study designs [22]. Controlled experiments reported higher C-reactive protein or other inflammatory markers during misalignment [16,17,21]. Repeated plaque imaging and long-term measurements linking these changes to subsequent coronary events were limited in the reviewed literature [12,13,14,15].

Studies of circadian hemostasis provided a separate evidence stream. Myocardial infarction onset displayed a morning peak in historical clinical observations [25], alongside morning increases in platelet aggregability [26]. Under controlled conditions, platelet activation peaked during the biological morning independently of scheduled behavior [23]. Plasminogen activator inhibitor-1 also showed an endogenous morning peak independent of the sleep–wake cycle [24]. These experiments established circadian variation in hemostatic markers but did not directly test whether night work precipitated plaque rupture.

Table 3 integrates the molecular and physiological pathways with their temporal role, supporting evidence, and limits of inference. Evidence levels use OCEBM 2011 [32]. The clinical-harms design framework is applied to observed health outcomes. L3 denotes nonrandomized controlled cohort/follow-up evidence. L5 denotes mechanism-based reasoning. Human experiments can establish surrogate changes without establishing ACS prevention. Accordingly, ACS extrapolations are identified separately from clinical associations. Levels are question-specific design guides, not formal certainty ratings. Indirectness, bias, inconsistency, and imprecision constrain interpretation. Acute and longer-term effects are not necessarily sequentially demonstrated within the same study. The domains correspond to the chronic substrate and acute trigger pathways in Figure 1. Figure 2 integrates the better-supported pathways into a double-hit model.

**Table 3:** Integrated molecular and physiological pathways relevant to coronary risk in shift workers.

Domain	Acute Disturbance	Longer-Term Implication	Measured Correlate	Evidence Strength ACS Directness	Directness to ACS Outcome
Clock and endocrine timing [12,13,14,15,19]	Central/peripheral clock desynchronization; altered cortisol timing	Disrupted metabolic and vascular regulation	Circadian phase; cortisol profiles; clock-gene expression	Human and preclinical mechanistic studies; low	Indirect/mechanistic; ACS inference L5
Autonomic balance [18]	Sympathetic activation and altered vagal modulation	Repeated cardiovascular strain	Heart rate; heart-rate variability	Human physiological studies; surrogate/mechanistic; low	Indirect/mechanistic; ACS inference L5
Hemodynamic load [16,17,52,53,54]	Higher blood pressure; altered sleep-related dipping	Hypertension associated with sustained exposure	Ambulatory/home blood pressure	Experimental and cohort studies; moderate for hypertension	Intermediate clinical harm for hypertension; indirect for ACS. Hypertension harms L3; ACS inference L5
Metabolic regulation [19,20,21,48,49,50,51,55,56,57,58]	Impaired glucose tolerance/insulin sensitivity; altered energy expenditure	Diabetes, adiposity and dyslipidemia associations	Glucose; HbA1c; lipids; waist circumference	Human experiments plus cohorts; moderate for metabolic harms	Intermediate clinical metabolic harms; indirect for ACS. Clinical metabolic harms L3; ACS inference L5
Inflammation and endothelium [16,17,21,27,28,29]	Increased inflammatory markers during misalignment	Possible contribution to endothelial dysfunction and plaque vulnerability	CRP; cytokines; endothelial function	Human surrogate outcomes plus atherosclerosis biology; mechanistic; low	Indirect/mechanistic; ACS inference L5
Oxidative stress [22,29]	Altered oxidant–antioxidant balance	Potential loss of nitric oxide availability and vascular injury	Oxidative-stress biomarkers	Heterogeneous observational/mechanistic evidence; low	Indirect/mechanistic; ACS inference L5
Thrombosis and fibrinolysis [23,24,25,26]	Biological-morning peaks in platelet activation and PAI-1	Potential trigger interaction with existing coronary disease	Platelet assays; PAI-1; symptom timing	Human circadian experiments and clinical timing studies; mechanistic/surrogate	Indirect/mechanistic for shift-work ACS; ACS inference L5
Sleep deficiency [8,20,35,60]	Short/fragmented daytime sleep; fatigue and sleepiness	Persistent sleep restriction; shift-work disorder	Sleep diary; actigraphy; validated symptom scales	Human sleep studies and post-ACS cohort; moderate for recurrent events	Post-ACS recurrent-event evidence; indirect for incident ACS. L3 for recurrent events; L5 for ACS prevention

Abbreviations: CRP, C-reactive protein; HbA1c, glycated hemoglobin; PAI-1, plasminogen activator inhibitor-1; BMAL1, brain and muscle ARNT-like 1; CLOCK, circadian locomotor output cycles kaput; PER, period; CRY, cryptochrome.

### 3.5. Behavioral and Occupational Correlates

Reviews and observational studies described differences in eating patterns, alcohol use, smoking, caffeine intake, exercise, and adiposity across shift schedules [61,62,63,64]. Findings for diet were heterogeneous, particularly regarding total energy intake, whereas meal timing and overnight food availability were recurrent concerns [61]. Lifestyle mediation analyses examined obesity and diabetes rather than ACS [62]. Separately, meta-analyses associated job strain with CHD and long working hours with incident CHD and stroke [65,66]. These exposures often accompanied night work but were not interchangeable measures of circadian misalignment. Consensus reviews also documented considerable variation in shift-work tolerance by individual characteristics and social circumstances [6,67].

## 4. Discussion

### 4.1. Circadian Misalignment and Multioscillator Desynchrony

The suprachiasmatic nucleus coordinates daily rhythms through retinal light input, while peripheral clocks also respond to feeding, activity, hormones, and temperature [7,12,13]. Night work can therefore move behavioral demands away from the internal phase that normally supports them. Because tissue clocks adapt at different rates, shifting sleep and meals may produce internal desynchrony rather than a coherent reversal of day and night. Circadian misalignment and insufficient sleep overlap, but neither fully represents the other. Their separation in human experiments supports considering both exposures when interpreting cardiovascular risk [15,19,20,21,48,49].

Over years, repeated metabolic, autonomic, and inflammatory disturbances could contribute to a susceptible coronary substrate. Impaired insulin sensitivity and glucose handling may promote diabetes and vascular injury, while adverse lipid profiles and adiposity increase established atherosclerotic risk [20,21,48,49,50,51,52,53,54,55,56,57,58,59]. Altered sleep and sympathetic regulation may sustain blood-pressure load and reduce recovery during sleep [16,17,18,52,53,54]. Meal timing provides a plausible connection between work schedules and peripheral metabolic clocks, although food quality, energy balance, and eating phase remain distinct influences [10,48,49,50,61]. These pathways are compatible with the observed cardiometabolic associations; their relative contributions to shift-related ACS have not been quantified.

Inflammation and oxidative stress may connect these disturbances to endothelial dysfunction and plaque vulnerability. Reduced nitric oxide availability, lipid oxidation, and inflammatory cell recruitment are established components of vascular pathology [27,28,29]. Circadian and sleep disruption could amplify these processes in workers with smoking exposure, diabetes, hypertension, or dyslipidemia [12,13,14,15,22]. However, a transient post-shift biomarker elevation does not demonstrate persistent vascular injury. C-reactive protein also reflects adiposity, infection, smoking, and psychosocial stress, and its interpretation depends on sampling time. Table 3 integrates the molecular pathways with their temporal role, supporting evidence, and limits of inference.

An acute trigger may then act on an existing substrate. Sympathetic activation, increased myocardial demand, altered vascular tone, and reduced fibrinolytic capacity could lower the threshold for an event [23,24,25,26,27,28]. Type 1 myocardial infarction involves acute atherothrombosis, whereas severe physiological stress can produce type 2 myocardial infarction through oxygen supply-demand imbalance [1,2]. The latter mechanism is related to acute ischemic events but should not be equated with plaque rupture. The same work episode could impose different consequences depending on coronary disease, blood pressure, hypoxemia, workload, and treatment status.

The biological morning deserves attention because circadian hemostatic rhythms need not track local clock time in a partially adapted worker. Behavioral transitions may occur at different internal phases during nights, recovery days, or return to daytime activity. Figure 3 summarizes this temporal framework. It should be read as a schematic of established physiological rhythms and proposed consequences of misalignment, rather than a measured event-risk curve for shift workers. Direct tests require circadian phase, recent schedule history, and event timing within the same participants.

### 4.2. Integrating Chronic Substrate and Acute Triggering

The evidence supports an association between shift work and coronary risk. The basis for claims specifically about ACS is narrower. Prospective observations establish temporal associations. Controlled experiments show that abnormal timing can alter relevant physiology. Together, these findings support the proposed double-hit model. This model separates chronic substrate formation from acute triggering. Figure 2 presents this model. It is an interpretive framework rather than a pathway established from work exposure through to coronary events within one study.

### 4.3. Schedule, Biological Susceptibility, and Social Context

Exposure duration alone cannot capture the biological burden of shift work. Night frequency, consecutive nights, shift length, rotation direction, intershift recovery, and schedule predictability determine opportunities for sleep and adaptation [6,7]. A fixed night schedule may permit partial adaptation, yet daylight exposure and daytime obligations can repeatedly reverse that adaptation. Rapid rotation provides little time for realignment, while short recovery intervals directly constrain sleep opportunity. Duration-response associations support considering cumulative exposure, but do not identify a universally safe roster or a threshold below which coronary risk disappears [9,10,37].

Chronotype and age may influence tolerance without providing reliable individual predictions of ACS. Evening preference can make some night schedules easier to accommodate, although social obligations and light exposure still limit adaptation [67]. Aging can reduce sleep consolidation and circadian amplitude while increasing baseline cardiometabolic burden. Apparent resilience among long-tenured older workers may also reflect selection, because less tolerant workers leave night schedules. Consequently, age or chronotype alone should not substitute for information about realized sleep, symptoms, recovery, and clinical risk.

Sex, gender, and reproductive circumstances require similar caution. Occupational segregation and unequal caregiving demands can produce different exposure patterns even when nominal rosters match [6,67,68]. Hormonal status and menopause are plausible biological modifiers, but current coronary evidence does not establish a consistently larger shift-work effect in one sex [36,67]. Pregnancy is a specific evidence gap: this review does not establish a pregnancy-by-shift-work interaction for ACS. Pregnancy-related changes in sleep, workload tolerance, and cardiovascular physiology warrant dedicated study, with obstetric outcomes kept distinct from coronary outcomes. Research should measure pregnancy and reproductive history directly rather than infer susceptibility from sex alone.

Pre-existing hypertension, diabetes, obesity, dyslipidemia, OSA, or coronary disease may reduce tolerance to repeated autonomic and metabolic stress. The hypertension subgroup finding in the Japanese cohort is compatible with this possibility, but remains insufficient to define a validated susceptibility rule [41]. OSA may add intermittent hypoxemia and sympathetic activation to restricted or irregular sleep. A positive OSA-screening result, however, is not equivalent to a confirmed diagnosis [35]. Genetic variants and circadian biomarkers likewise remain research measures rather than validated tests for occupational coronary-risk selection.

Workplace context determines additional exposures. Healthcare work may combine nights with emotional demand, lifting, interrupted breaks, and staffing pressure; transport work may add prolonged sitting and safety-critical responsibility. Industrial schedules may coincide with noise, heat, particulates, or chemical exposure [6,12,68]. Housing conditions, commuting, income insecurity, caregiving, and access to care further shape recovery. These factors can confound or mediate schedule associations, and may amplify susceptibility. Table 4 groups proposed risk and protective factors while distinguishing general health associations from evidence of direct circadian or ACS effects.

### 4.4. Clinical Meaning and the Boundary of Prevention Claims

For clinical interpretation, shift work is best treated as a potentially contributory exposure embedded within established cardiovascular risk. Its relevance may be greatest when demanding schedules coincide with poor sleep, uncontrolled risk factors, or existing coronary disease. The observational post-ACS evidence supports taking an occupational history, but does not show that continuing night duty caused recurrence or that stopping nights improves prognosis [35]. Similarly, associations with long working hours concern a related occupational exposure and should not be presented as proof about post-ACS return-to-work schedules [66].

The proposed pathways suggest potentially modifiable targets, but improved sleep or biomarkers cannot be assumed to prevent ACS. A treatment can improve alertness without restoring internal alignment, and a phase-shifting intervention may have different effects under fixed and rapidly rotating schedules [13,15]. Sleep opportunity, sleep quality, circadian phase, and cardiovascular endpoints therefore require separate assessment. Standard prevention remains the clinical foundation, while schedule and behavioral interventions address additional burdens whose coronary benefits still need outcome evaluation [1,14,71]. Section 5 translates this distinction into practical clinical and workplace actions.

Risk and protective factors also need careful causal language. Smoking, poor diet, inactivity, stress, and metabolic disease may precede shift work, develop partly because of its constraints, or interact with it [59,61,62,63,64,65]. Their position in the pathway changes what statistical adjustment estimates. Moreover, an absent association within current smokers does not imply protection from smoking; subgroup imprecision and differences in baseline risk remain alternative explanations [36]. Proposed protective behaviors should therefore be described by their demonstrated sleep, metabolic, or general cardiovascular effects, with direct prevention of shift-related ACS reserved for evidence that measures that outcome.

### 4.5. Limitations of the Evidence and This Review

Residual confounding limits causal attribution across occupational cohorts. Shift and day workers differ in socioeconomic position, workload, job control, environmental exposures, health behaviors, and access to care [6,37,44,47]. Incomplete adjustment may overestimate effects attributable to work timing. Conversely, adjustment for blood pressure, adiposity, or diabetes can remove part of a mediated effect. Studies estimating total occupational effects and those estimating effects independent of cardiometabolic mediators consequently answer different questions. Most clinical trials do not assess whether patients work day or night. This limits inference about the contribution of work timing to trial outcomes.

Healthy-worker selection can obscure harmful associations. Workers who develop sleep intolerance, hypertension, or other illness may move to daytime schedules, leaving a selected group among continuing night workers [6,37]. Classifying former shift workers as unexposed can further dilute contrasts. Baseline exposure measurements also fail to capture changes during follow-up. These problems are especially relevant to cumulative-duration analyses, where surviving and remaining in a particular job are prerequisites for high exposure.

Endpoint heterogeneity constrains the manuscript’s ACS focus. Broad CVD composites, CHD diagnoses, ischemic heart disease mortality, MI/angina, and recurrent coronary composites are related but not interchangeable outcomes [8,9,10,33,34,35,36,37,38,39,40,41,42,43,44,45,46,47]. Angina without adjudication may include stable disease, while coronary revascularization is influenced by clinical investigation and treatment decisions. The reviewed studies rarely separated type 1 from type 2 myocardial infarction or linked event timing to the immediately preceding roster. Thus, evidence for chronic coronary risk is stronger than evidence assigning an acute event to a particular shift.

Mechanistic evidence has a different limitation. Small, short experiments can isolate circadian or sleep effects, but frequently involve selected participants under conditions unlike long-term employment [16,17,18,19,20,21,48,49,50,51]. Biomarkers and autonomic measurements are not validated substitutes for ACS outcomes. Cross-sectional vascular studies cannot establish whether misalignment preceded vascular injury, and differences in sampling phase can create apparent between-group differences [22,40,43]. Molecular evidence supports plausibility, but should not be presented as proof that every pathway operates in every worker.

Finally, this narrative review used targeted searches and qualitative synthesis rather than an exhaustive systematic-review design. The representative study tables do not constitute a complete inventory, and no new pooled estimate was calculated. Selection and interpretive bias may remain despite explicit eligibility and appraisal criteria. Overlapping cohorts across reviews also mean that multiple meta-analyses are not fully independent replications. These limitations support a bounded conclusion: the literature identifies a plausible, potentially modifiable contribution to coronary risk, while the magnitude of an ACS-specific causal effect remains uncertain.

### 4.6. Research Directions That Address These Uncertainties

Future cohorts should combine electronic rosters with repeated sleep, light, and meal-timing measurements. A common exposure dataset should capture shift times, night frequency, consecutive nights, rotation, shift duration, recovery intervals, overtime, cumulative exposure, and time since leaving night work [6,7]. Nested circadian-phase measurements could distinguish biological misalignment from insufficient sleep. Outcome adjudication should separate STEMI, NSTEMI, unstable angina, type 1 and type 2 myocardial infarction, and recurrent events. This combination would connect schedule-based exposure with realized biology and clinically specific outcomes.

Analyses should model exposure and mediators over time, address health-related schedule changes, and state whether they estimate total or direct effects. Natural experiments involving roster redesign or transitions from night to day work can strengthen causal inference. Mechanistic follow-up should test whether within-person improvements in alignment precede changes in blood pressure, glucose regulation, endothelial function, inflammation, or platelet activity. Standardizing sampling relative to both clock time and internal phase would reduce ambiguity about transient versus persistent effects [14,15].

Intervention studies should evaluate feasible workplace packages and prospectively record adherence, recovery, symptoms, and unintended effects. Larger studies or linked registries are needed to establish whether intermediate improvements translate into fewer coronary events. After ACS, comparisons of phased return, temporary daytime assignment, and reduced schedule intensity should include recurrent events, quality of life, and work retention. Recruitment should deliberately represent older workers, both sexes, pregnancy and reproductive circumstances where relevant, lower-income occupations, and established coronary disease [67,68,74]. These designs would resolve specific uncertainties while testing interventions in the settings where workers must use them.

## 5. Clinical Implications and Recommendations

### 5.1. Cardiovascular Assessment and Sleep Health

The cited ACS and prevention guidelines do not provide a validated shift-work risk score [1,71]. Work history should therefore supplement conventional assessment. Clinicians can document night/rotating/on-call duty, consecutive nights, shift length, quick returns, cumulative exposure, commuting, and recovery opportunity. The history should also cover workday and rest-day sleep, insomnia, excessive sleepiness, snoring or witnessed apnea, stimulant use, and medication adherence. These questions identify potentially modifiable constraints without assuming that every exposed worker has a circadian disorder.

Blood pressure, lipids, glycemia, adiposity, smoking, physical activity, family history, and ischemic symptoms remain central to prevention. Home or ambulatory blood-pressure measurements can clarify suspected masked hypertension or abnormal dipping. Screening intervals should reflect baseline risk and symptoms rather than an unvalidated schedule-specific rule. Occupational programs need accessible appointments and treatment follow-up for night staff; identifying abnormalities without providing care is unlikely to improve health.

Sleep duration, quality, and timing deserve clinical attention [14,72]. Protected sleep opportunity, control of daytime light and noise, and evaluation of symptomatic obstructive sleep apnea (OSA) or shift-work disorder are reasonable measures [8,60,75]. Timed light, darkness, sleep scheduling, and selected use of melatonin target diagnosed sleep or alertness problems [76,77]. Evidence for these outcomes should not be presented as evidence that circadian treatment prevents myocardial infarction. Table 5 separates practical actions from their evidential limitations.

### 5.2. Moderate Physical Activity

Regular moderate physical activity remains part of cardiovascular prevention and rehabilitation [1,71]. For shift workers, it may additionally act as a nonphotic timing cue, influencing activity–rest patterns, sleep pressure, body temperature, and peripheral metabolic rhythms. Easton and colleagues discuss how the timing, intensity, duration, and type of activity could be used to facilitate shift-work adaptation [70]. Their article is a Perspective integrating existing evidence and proposing research directions, rather than a trial demonstrating circadian realignment or fewer cardiovascular events in shift workers.

Practical measures include walking or other moderate activity during feasible waking periods, breaking up prolonged sitting, and providing accessible activity opportunities for night staff. Timing should be matched to the roster, intended sleep, fatigue, and individual tolerance. Exercise performed at different biological phases may shift rhythms in different directions; a universal clock-time prescription is therefore premature. Activity should not displace recovery sleep, and post-ACS exercise should follow rehabilitation advice. Combining moderate activity with stable sleep, light, and meal routines is plausible, but its additional circadian benefit requires direct testing.

### 5.3. Mediterranean-Style Diet and Chrononutrition

Mediterranean-style eating emphasizes vegetables, fruit, whole grains, legumes, nuts, and unsaturated fats, with fish and limited highly processed foods. Patel and Cheung’s scoping review examined the Mediterranean diet and chrononutrition in relation to sleep quality [69]. Much of the evidence was observational and derived from populations other than shift workers. Better diet quality and more favorable meal timing may support sleep and metabolic health, but the review does not establish that Mediterranean eating restores circadian phase or prevents shift-work-related ACS.

The relevant clinical distinction is between food composition and timing. Shift workers may have reasonable total energy intake while eating irregularly or relying on large meals during the biological night [61]. Workplace food provision can support Mediterranean-style choices and planned meals, with smaller overnight portions when practical. Timing should accommodate medication, diabetes management, energy needs, and sleep opportunity; rigid fasting windows are not justified by this evidence. Potential pathways include improved postprandial glucose handling and lower inflammatory burden, while regular feeding may provide more consistent peripheral timing cues. These mechanisms remain hypotheses when applied specifically to ACS prevention in shift workers.

### 5.4. Mindfulness and Other Proposed Protective Factors

Mindfulness can be considered an adjunct for sleep-related distress and psychological stress. In a randomized trial among 49 older adults with sleep disturbances, Black and colleagues compared a six-week mindfulness-awareness program with sleep-hygiene education and reported greater improvement in sleep quality and selected daytime symptoms [73]. Participants were not shift workers, and the trial did not test circadian phase correction or cardiovascular events. Reduced cognitive arousal and improved management of stress may facilitate recovery sleep, but that proposed route should be distinguished from demonstrated entrainment of the circadian clock.

Brief mindfulness practice may be offered according to preference and feasibility, alongside assessment of insomnia, OSA, or mental-health symptoms. It should not replace indicated treatment or workplace changes. Table 4 includes the broader proposed protective factors. Evidence for yoga, prayer, or prolonged mastication as interventions for shift-work-induced circadian dysfunction or ACS prevention is insufficient in the reviewed material. Alcohol moderation means reducing excessive intake; abstainers should not start drinking for presumed cardiovascular benefit. Supporting sleep, diet, activity, and stress management may improve health, but the available evidence does not justify claiming that every listed factor prevents CVD by correcting circadian misalignment.

### 5.5. Secondary Prevention and Return to Work After ACS

After ACS, indicated antiplatelet therapy, lipid lowering, blood-pressure and diabetes control, smoking cessation, rehabilitation, and adherence remain foundational [1]. The association between overnight-work history and later major coronary events in a post-ACS cohort supports asking about work exposure [35]; it does not prove that stopping nights reduces recurrence. Medication plans should accommodate actual waking and sleeping times and avoid missed or duplicate doses when schedules change.

Return-to-work decisions should integrate symptoms, ventricular function, residual ischemia, arrhythmia, functional capacity, comorbidity, job demands, fatigue-related safety risk, and recovery opportunity. A phased return, temporary day assignment, fewer consecutive nights, shorter shifts, or avoidance of quick returns may be considered when feasible. No trial-derived rule requires all patients with ACS to permanently stop night work. Persistent angina, decompensated heart failure, uncontrolled blood pressure, clinically important arrhythmia, or severe untreated sleepiness warrants stabilization and individualized review before demanding or safety-critical duty. Cardiology, primary care, sleep medicine, and occupational health should coordinate reassessment while considering employment and worker preferences.

### 5.6. Workplace Implementation and Evaluation

Roster design determines whether individual advice is achievable. Consensus recommendations favor limiting consecutive nights and shift length, protecting at least 11 h between shifts, and considering no more than three consecutive nights and night shifts of no more than nine hours where feasible [74]. These are recommendations derived from mixed health and safety evidence, not validated ACS thresholds. Predictable schedules, worker participation, and adequate staffing support recovery; adjustments should also consider income, caregiving, commuting, and the possibility of second-job exposure [68].

Healthy food, protected breaks, smoking-cessation support, accessible exercise opportunities, and care outside conventional office hours address practical barriers [61,62,63,64,78]. Figure 4 presents an evidence-informed assessment pathway, and Table 6 identifies emerging interventions and their limits. Implementation should measure roster exposure, sleep, fatigue, blood pressure, metabolic outcomes, adverse effects, and work retention. Longer follow-up is needed for adjudicated cardiovascular events. Improvements in sleep or biomarkers should not automatically be interpreted as reductions in ACS risk.

The algorithm is intended for use in primary care, cardiology, and occupational health settings. It guides clinicians through screening, comprehensive evaluation, risk stratification, and tiered interventions. High-risk features include recent acute coronary syndrome, uncontrolled hypertension, severe untreated obstructive sleep apnea, decompensated heart failure, frequent angina, and poorly controlled diabetes. Management recommendations are evidence-informed but should be individualized based on patient preferences, job demands, and organizational feasibility. Abbreviations: ACS, acute coronary syndrome; AHA, American Heart Association; AHI, apnea-hypopnea index; BP, blood pressure; CPAP, continuous positive airway pressure; ESC, European Society of Cardiology; OSA, obstructive sleep apnea.

## 6. Conclusions

Night and rotating shift work are associated with a modestly higher risk of coronary heart disease and myocardial infarction, and limited direct evidence also links shift work to incident and recurrent ACS. The relationship is biologically plausible and displays duration-response features, but heterogeneous exposure definitions, residual confounding, healthy-worker selection, and the scarcity of ACS-specific studies preclude a simple causal estimate.

The most coherent explanation is a double-hit model: chronic circadian misalignment and sleep disruption build a susceptible metabolic, inflammatory, endothelial, and atherosclerotic substrate, while acute autonomic, hemodynamic, and prothrombotic changes can trigger ischemia or thrombosis. Schedule design, chronotype, age, comorbidity, sleep disorders, occupation, and social context determine the realized dose. Clinical practice should therefore integrate work schedule and sleep health into standard cardiovascular assessment, especially after ACS. Unproven circadian therapies should not substitute for guideline-directed secondary prevention. Organizational control of consecutive nights, shift duration, quick returns, recovery opportunity, and access to healthy working conditions offers the most defensible preventive strategy while outcome trials are developed. More prospective ACS-specific studies are needed before stronger causal or generalizable conclusions can be made.

## Figures and Tables

**Table 1:** Shift-work exposure dimensions relevant to ACS research.

Exposure Dimension	Operational Measures	Relevance
Clock timing	Start/end time; overlap with 00:00–05:00; early starts	Determines work at adverse circadian phases and timing of acute triggers.
Pattern	Permanent night; rotating; evening; split; irregular/on-call	Different patterns produce different adaptation, sleep loss, and social jet lag.
Intensity	Night shifts/month; nights/year; on-call activations	Separates occasional exposure from recurrent biological-night work.
Sequence	Consecutive nights; forward/backward rotation; speed	Shapes cumulative sleep debt and repeated phase advances/delays.
Recovery	Hours between shifts; quick returns; days off; commute	Constrains sleep opportunity and autonomic/metabolic recovery.
Duration	Hours/shift; overtime; cumulative years; time since cessation	Captures acute workload and long-term atherogenic exposure.
Realized biology	Sleep duration/regularity; light; meal timing; circadian phase	Explains why identical rosters may produce different physiological doses.

**Table 4:** Risk-related factors, biological susceptibility and candidate protective factors relevant to circadian misalignment.

Behavioral, Psychosocial or Environmental Factors	Biological Conditions or Susceptibility Markers	Candidate Protective Factors
Western dietary pattern	Hypertension	Mediterranean-style diet [69]
Tobacco use	Diabetes mellitus	Moderate physical activity [70]
Harmful alcohol use/alcohol dependence	Coronary artery disease	Moderation of excessive alcohol intake [63,71]
Mental disorders	Metabolic syndrome	Reducing mental stress [65]
Sleep disorders	Aging	Adequate, regular and restorative sleep [60,72]
Sedentary behavior	Undernutrition	Meditation [73]; yoga and prayer *
Pollution indoors and at work (environmental)	Hyperuricemia	Thorough mastication during eating *

This conceptual inventory is not a validated causal classification. The columns are independent lists, not matched causal or therapeutic pairs. Comorbidities may increase vulnerability or coexist with misalignment rather than cause it. Evidence chiefly concerns conventional cardiovascular risk, sleep, or coping [14,55,56,57,58,59,61,62,63,64,65,69,70,71,72,73]. Direct shift-worker circadian or ACS benefits remain unproven. * No direct supporting evidence for yoga, prayer, or mastication was identified in the cited intervention literature. Alcohol advice concerns reducing harmful intake; it does not recommend initiating drinking.

**Table 5:** Evidence-informed clinical and workplace actions.

Domain	Evidence-Informed Action	Evidence Basis	Important Limitation
Clinical assessment	Record schedules, cumulative exposure, recovery and sleep symptoms.	Exposure frameworks and coronary cohorts [6,32,33]	No validated shift-work ACS risk score.
Established prevention	Control blood pressure, lipids, glycemia, smoking and weight; support adherence and cardiac rehabilitation.	ACS/CVD guidelines [1,71]	Does not remove circadian exposure.
Physical activity	Offer moderate exercise and activity breaks compatible with sleep and clinical capacity.	Guideline prevention; shift-work Perspective [70,71]	Optimal phase-specific exercise prescription unproven.
Diet and meal timing	Provide Mediterranean-style food across shifts; support feasible, consistent meal timing.	Eating-pattern and sleep reviews [61,69]	No demonstrated circadian realignment or ACS benefit in shift workers.
Sleep and stress	Assess sleep disorders; offer indicated treatment and optional mindfulness support.	Sleep guidance; older-adult mindfulness RCT [8,60,72,73,75]	Mindfulness evidence is indirect for shift workers.
Work organization	Use predictable rosters, adequate recovery, protected breaks, and night-accessible preventive services.	Working-time consensus and intervention review [68,74,76,78]	Evaluate feasibility, uptake, and health outcomes.
Circadian support	Individualize light, darkness, naps, and any melatonin use through clinical assessment.	The circadian/sleep literature [60,75,76,77]	Timing matters; no proven ACS prevention.
After ACS	Individualize return to work; consider day duty, fewer nights and phased hours.	Post-ACS association and clinical assessment [35]	No universal roster rule or randomized outcome evidence.

**Table 6:** Emerging interventions for mitigating cardiovascular risk in shift workers: evidence, limitations, and implementation considerations.

Intervention	Rationale	Evidence	Limitations and Implementation
Roster redesign	Protect recovery and reduce repeated sleep loss.	Consensus informed by health/safety research [68,74].	No ACS trial; negotiate schedules, worker preferences, and adequate staffing.
Moderate physical activity	Possible nonphotic timing cue; supports alertness, mood, and fitness.	Easton et al.: Perspective synthesizing laboratory and observational work [70].	Timing/light may alter phase-shift direction; individualize exercise without compromising sleep. Occupational circadian efficacy is unconfirmed.
Mediterranean-style diet	Improve nutrient quality; potentially support sleep and cardiometabolic health.	Patel and Cheung: 33 studies (24 dietary-pattern, 9 chrononutrition); predominantly observational sleep evidence [69].	Associations do not establish realignment; provide affordable healthy food on every shift.
Meal-timing interventions	Reduce misalignment between eating and metabolic rhythms.	Shift-worker eating-pattern review; mixed chrononutrition sleep findings [61,69].	A specific eating window is not established by these reviews; test practical timing with individual clinical needs.
Mindfulness	Address sleep-related cognitive/emotional arousal.	Black et al.: 49 adults aged ≥ 55 years; six-week RCT improved sleep and daytime impairment [73].	No shift-worker, circadian-phase, or CVD endpoint; offer as an optional adjunct and monitor symptoms.
Timed light/darkness	Modify circadian phase and alertness.	Circadian intervention guidance [76,77].	Match timing to roster/chronotype; avoid unwanted phase shifts. Cardiovascular-event benefit is untested.
Melatonin	Sleep-promoting and phase-shifting effects.	Sleep-disorder practice parameters and review [60,77].	Clinical review of timing, safety, and interactions is required; no ACS-prevention indication.
Planned naps	Reduce sleep pressure and support alertness.	The sleep/shift-work literature [60].	Provide suitable space and allow recovery from sleep inertia before safety-critical work.
Adapted CBT-I	Improve insomnia-related sleep behaviors and distress.	The clinical shift-work literature [60,75].	Schedule-specific adaptation and access to trained providers are needed; no cardiovascular endpoint evidence.
Workplace health promotion	Improve access to smoking cessation, healthy food, and physical activity.	Behavioral studies and intervention review [61,62,63,64,78].	Ensure access during nights; measure uptake, sustained behavior change, and clinical outcomes.

Evidence concerns the studied sleep, behavioral, or physiological outcomes. None of the three newly added references establish ACS prevention in shift workers. Abbreviations: CBT-I, cognitive-behavioral therapy for insomnia; RCT, randomized controlled trial.

## Data Availability

No new data were created or analyzed in this study. Data sharing is not applicable to this article.

## Abbreviations

The following abbreviations are used in this manuscript:

ACS	Acute Coronary Syndrome
AHA	American Heart Association
AHI	Apnea–Hypopnea Index
BMI	Body Mass Index
BP	Blood Pressure
CHD	Coronary Heart Disease
CPAP	Continuous Positive Airway Pressure
CV	Cardiovascular
CVD	Cardiovascular Disease
ESC	European Society of Cardiology
FMD	Flow-Mediated Dilation
HbA1c	Glycated Hemoglobin (Hemoglobin A1c)
HDL	High-Density Lipoprotein
HOMA-IR	Homeostatic Model Assessment for Insulin Resistance
HR	Hazard Ratio
HRV	Heart-Rate Variability
Hs-CRP	High-Sensitivity C-Reactive Protein
ICAM-1	Intercellular Adhesion Molecule-1
IHD	Ischemic Heart Disease
IL-6	Interleukin-6
LV	Left Ventricular
MI	Myocardial Infarction
NSTEMI	Non-ST-Segment-Elevation Myocardial Infarction
OSA	Obstructive Sleep Apnea
PAI-1	Plasminogen Activator Inhibitor-1
RR	Relative Risk
STEMI	ST-Segment-Elevation Myocardial Infarction
TG	Triglycerides
TNF-α	Tumor Necrosis Factor-Alpha
VCAM-1	Vascular Cell Adhesion Molecule-1
